# Supplementary material for: A high-resolution mRNA expression time course of embryonic development in zebrafish
Source: eLife. 2017 Nov 16;6:e30860. doi: 10.7554/eLife.30860 (PMC5690287; doi:10.7554/eLife.30860)
Supplement: Supplementary file 6. [file elife-30860-supp6.zip › biolayout-clusters-files/Cluster232-genes.html]

Cluster232


# Cluster232: Genes

| Ensembl ID | Gene Name | Chr | Start | End | Biotype |
| --- | --- | --- | --- | --- | --- |
| ENSDARG00000074221 | ENSDARG00000074221 | 11 | 5641816 | 5713549 | protein\_coding |
| ENSDARG00000022176 | IGLON5 | 16 | 50876567 | 50960056 | protein\_coding |
| ENSDARG00000087402 | TPM1 | 25 | 32707260 | 32764898 | protein\_coding |
| ENSDARG00000057000 | camkvl | 23 | 20637436 | 20710397 | protein\_coding |
| ENSDARG00000033447 | nxph1 | 19 | 38270784 | 38340374 | protein\_coding |
| ENSDARG00000063310 | oxr1b | 19 | 7390120 | 7488008 | protein\_coding |
